# Localized Ambient Solidity Separation Algorithm Based Computer User Segmentation

**DOI:** 10.1155/2015/829201

**Published:** 2015-06-28

**Authors:** Xiao Sun, Tongda Zhang, Yueting Chai, Yi Liu

**Affiliations:** ^1^National Engineering Laboratory for E-Commerce Technology, Tsinghua University, Beijing 100084, China; ^2^DNSLAB, China Internet Network Information Center, Beijing 100190, China; ^3^Electrical Engineering Department, Stanford University, Stanford, CA 94305, USA

## Abstract

Most of popular clustering methods typically have some strong assumptions of the dataset. For example, the *k*-means implicitly assumes that all clusters come from spherical Gaussian distributions which have different means but the same covariance. However, when dealing with datasets that have diverse distribution shapes or high dimensionality, these assumptions might not be valid anymore. In order to overcome this weakness, we proposed a new clustering algorithm named localized ambient solidity separation (LASS) algorithm, using a new isolation criterion called centroid distance. Compared with other density based isolation criteria, our proposed centroid distance isolation criterion addresses the problem caused by high dimensionality and varying density. The experiment on a designed two-dimensional benchmark dataset shows that our proposed LASS algorithm not only inherits the advantage of the original dissimilarity increments clustering method to separate naturally isolated clusters but also can identify the clusters which are adjacent, overlapping, and under background noise. Finally, we compared our LASS algorithm with the dissimilarity increments clustering method on a massive computer user dataset with over two million records that contains demographic and behaviors information. The results show that LASS algorithm works extremely well on this computer user dataset and can gain more knowledge from it.

## 1. Introduction

*Background and Related Work*. The fast growing Internet technologies and multidisciplinary integration, such as social network, e-commerce, and bioinformatics, have accumulated huge amounts of data, which is far beyond human beings' processing ability from both data scalability and structure complexity [1]. For example, as scientists study the working mechanism of the cell, they would gather data about protein sequences or genomic sequences, which could be as large as tens or hundreds of terabyte and have a fairly intricate structure inside. Even the smartest person has no way to deal with such a dataset without any assistant tool. Data mining technologies [2] like semisupervised learning [3] and deep learning [4] are developed to address this problem and play an important role in a lot of fields, such as smart home [5], supporting decision system [6], biology [7], and marketing science [8]. In most of these areas, people constantly want to gain knowledge and learn structure from the data they collected. Clustering [9], as one of the most important unsupervised learning methods in data mining, is designed for finding hidden structure in unlabeled dataset, which can be used for further processing, such as data summarization [10] and compression [11].

Despite the dozens of different clustering methods from a variety of fields, they can be roughly divided into two categories, partitional method and hierarchical method [12]. Partitional clustering method tries to generate definite numbers of clusters directly. Considering the computationally prohibitive cost to optimize criterion function globally, iterative strategy is usually adopted. On the other hand, hierarchical clustering method generates a group of clustering results; different threshold parameters lead to different clustering results. Both clustering methods have limitations which make them perform badly when applying on some dataset without any change like human behaviour dataset which has various kinds of features and scales in high-dimensional space. The first limitation is the dimensionality. The dataset we are dealing with is usually with a dimension higher than 3, which makes it almost impossible for people to have a clear intuition of the data distribution. Current clustering methods typically need a given parameter to decide the number of generated clusters. For example, in *k*-means [13], a predetermined parameter *k* which represents the number of clusters to be generated is required to run the algorithm. In single link and complete link [8], threshold parameter plays a similar role. In such cases, the selection of parameter is highly subjective judgement and will become harder as the dimension goes up. Also, high dimensionality makes traditional Euclidean density notion meaningless, since the density tends to 0 as dimensionality increases. Therefore, density-based clustering methods with traditional similarity would get into trouble. The second limitation is the diversity of data distribution shapes. The distribution of objects in dataset is typically diverse, which may involve isolated, adjacent, overlapping, and background noise at the same time. However, current clustering methods usually make some strong assumptions on data distribution shape. For example, *k*-means implicitly assumes circle shape of clusters because of its Euclidean distance based optimization function, which makes it perform badly when handling nonglobular cluster cases. Density-based clustering method can handle clusters of arbitrary shape, but it has difficulties in finding clusters if their densities vary a lot. Taking density-based spatial clustering of applications with noise (DBSCAN) as an example, its sensitivity of density variation is influenced by the indicated radius, which is fixed and selected in advance, so it would have troubles if the densities of clusters vary widely. In a word, since a lot of current massive datasets typically have high dimensionality and diverse distribution shapes, traditional clustering methods like *k*-means, single link, complete link, or basic density-based clustering algorithm are no longer a good choice. In this paper, we address the problem of clustering the dataset with high dimensionality and diverse distribution shapes and try to develop an applicable clustering algorithm.

For the validation of clustering algorithm in practical applications, a segmentation of Chinese computer users is carried out in this paper. Segmentation is another name of clustering in some specific area. For example, in computer version, image segmentation [14] means to partition a digital image into several segments to make it easier for understanding or further analysis. While in marketing management, market segmentation [15] or customer segmentation [16] uses clustering techniques to segment target market or customers into a small number of groups who share common needs and characteristics. The goal of market segmentation or customer segmentation is to address each customer effectively and maximize his value according to the corresponding segment. Related researches have been conducted about food market [17, 18], vegetable consumers [19], financial market [20], banking industry [21], flight tourists [22], rail-trail users [23], and so on. Although lots of works have been done about traditional offline market segmentation, not enough attention is given to computer user or online market segmentation. Additionally, existing researches about online market segmentation typically collect data through an online survey or questionnaire [16, 24, 25], which cannot ensure the accuracy and objectivity of responders' behavior information, such as computer use time per week and browsing time per week. In our research, computer users demographic information is self-administered, while their behaviour information is extracted from the log files of background software which real-timely records their human-computer interaction behaviours term by term. Therefore, the computer user behaviour information used in our research can minimize the error caused by subjective perception bias.

*Dataset*. The dataset used in this paper is provided by China Internet Network Information Center (CNNIC) [26], which recruits a sample of more than 30 thousand computer users and records more than ten million items per day about their computer interaction behaviour. These volunteers are required to install background software on their daily used online computers, by which their interaction behaviours will be collected. In addition to interaction behaviours, demographic information, such as gender and age, has also been collected when a volunteer creates his account. Thousands of personal attributes' information, together with their behaviour information, set up the validation foundation of our proposed algorithm.

More specifically, the data used in this paper are extracted from 1000 randomly selected volunteers' log files with over two million records in 7 days and their personal attribute information. To protect privacy, the volunteer's name is replaced by his hashed value so that actual identification cannot be retrieved.

*Outline of the Paper*. The remainder of the paper is organized as follows.  Section 2.1 shows the performance of a hierarchical dissimilarity increments clustering method on a designed two-dimensional benchmark dataset, and several drawbacks are pointed out; Sections 2.2 and 2.3 propose a new isolation criterion based on the nonhomogeneous density within a cluster; Section 2.4 demonstrates the performance of our LASS clustering algorithm on the previous two-dimensional benchmark dataset. In Section 3, our LASS clustering algorithm is applied on computer users dataset, which contains their demographic and behaviour information.  Section 3.1 describes the cleaning process of raw data and 7 features are extracted to characterize computer users; Sections 3.2 and 3.3 describe the data normalization process and define a dissimilarity measurement; in Section 3.4, our LASS algorithm is performed on the normalized dataset; segmentation and validation results are given; in Section 3.4, we give a comprehensive summarization and discussion of the segmentation results. Finally we draw conclusions of this paper and point out some potential directions in Section 4.

## 2. Dissimilarity Increments and Centroid Distance Criteria Based Clustering Method

Based on the dissimilarity increments between neighbouring objects within a cluster, a new isolation criterion called dissimilarity increments is proposed and a hierarchical agglomerative clustering algorithm is designed [27]. In this section, we first generate a two-dimensional benchmark dataset to test the effectiveness of the dissimilarity increments clustering method. Strengths and weaknesses of this method are discussed compared to other classical clustering methods. After that, in order to make up for the pointed drawbacks, we analysed the characteristics of density distribution within a cluster and proposed a new isolation criterion called centroid distance, based on which a nonhomogeneous density detection algorithm is designed to generate further subclusters from an isolated parent cluster. Then an integration of the original dissimilarity increments clustering method and our proposed centroid distance isolation criterion is made; a new clustering algorithm named localized ambient solidity separation (LASS) is developed. Finally, our LASS algorithm is applied on the two-dimensional benchmark dataset again and the performance is demonstrated.

### 2.1. Dissimilarity Increments Based Clustering Method

Integrating dissimilarity increments isolation criterion with hierarchical clustering method, a novel hierarchical agglomerative clustering method has been proposed [27], which is called dissimilarity increments clustering method in this paper. Compared with classical hierarchical clustering methods, such as single link or complete link, this method does not need a threshold to determine the number of clusters. Instead, the number of generated clusters is automatically decided by algorithm. While on the other hand compared with classical partitioning clustering methods such as *k*-means this method does not make any prior hypothesis about cluster shape and thus can handle clusters of arbitrary shape as long as they are naturally isolated.

**Figure 1:**
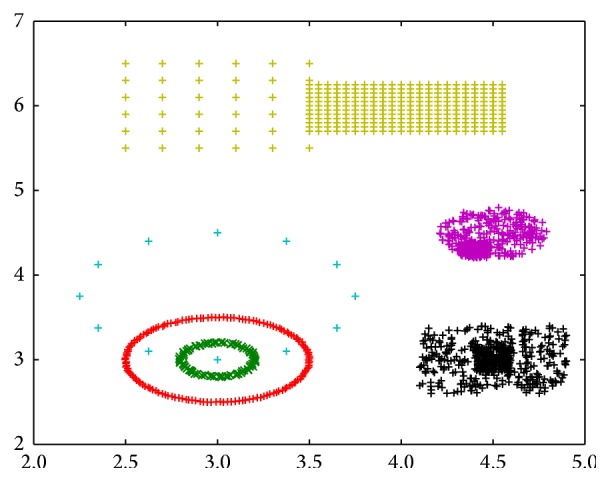
Result generated by dissimilarity increments clustering method.

However, dissimilarity increments clustering method also has some drawbacks. That is, due to the nature of hierarchical clustering method, it is not sensitive to the points in adjacent, overlapping, and background noise area. In Figure 1, a two-dimensional benchmark dataset is designed to show this fact. This dataset contains six well-isolated groups, three of which have nonhomogeneous internal structure. We use this dataset to test the performance of a clustering algorithm on identifying clusters when they are completely isolated and somewhat in touching. As we can see from the figure, the dissimilarity increments clustering method grouped the points into six clusters, which is consistent with first glance intuition. However, the clustering result also shows that this method is not applicable in three cases, which are the yellow cluster in the upper half of Figure 1 and the red and green clusters in the right half of Figure 1. The case of yellow forks represents two adjacent clusters, the case of red forks represents two overlapping clusters, and the case of green forks represents a cluster under background noise.

### 2.2. The Density Distribution within a Cluster

Considering the six identified clusters in Figure 1, we could find that the points' density distribution within a cluster could be quite different from one another. Specifically, the points' density of the three circle-shaped clusters in the bottom left part of Figure 1 is homogeneous, while the remaining three clusters are nonhomogeneous. Nonhomogeneous means that the points' density does not change continuously and smoothly but heavily with a clear boundary of two touching clusters. So a mechanism could be designed to identify potential subclusters within a given cluster based on the nonhomogeneous or heterogeneous distribution of density.

The first question is how to define and measure density. In convention, the concept of points' density refers to the number of points in unit area. But just as it is mentioned in Background and Related Work (see Section 1), Euclidean notation of density would have trouble with high-dimensional dataset and cannot identify clusters when their densities vary widely. The key idea to address these two problems is to associate density with each point and its surrounding context and, moreover, to associate isolation criterion with points' count distribution rather than absolute values. In this paper, the density around point *x*_*i*_ is defined as the reciprocal of the centroid distance of *x*_*i*_'s *n* nearest neighbours, just as formula (1) shows. In this formula, Distance(·) is a defined function to output the distance of two points, set *X* is a collection of *x*_*i*_'s *n* nearest neighbour points, *x*_*m*_ refers to the point which has the largest distance to *x*_*i*_ in set *X*, and Centroid(·) is a function to calculate the centroid point of a given point set. Intuitively, the point which lies in high density area will have a small centroid distance and thus have a large value of density around:

(1) Densityxi=1Centroid_Distancexi=1n−1×Distancexm,CentroidX−xm.

**Figure 2:**
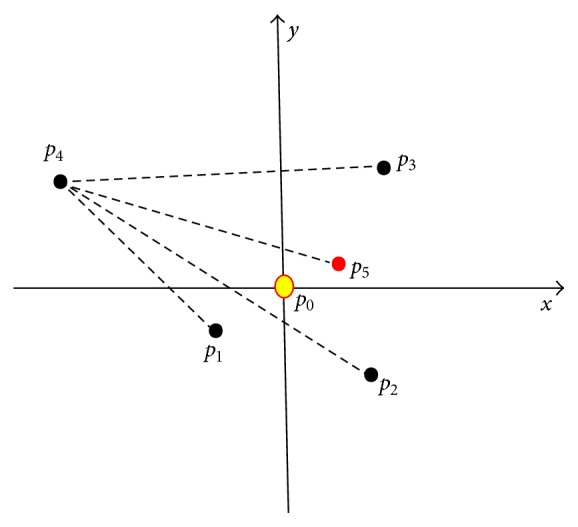
Centroid distance of point *p*_0_.

A more concrete example of centroid distance is the two-dimensional case shown in Figure 2, in which *p*_0_ is the target point and *p*_1_ ~ *p*_4_ are *p*_0_'s 4 nearest neighbour points among the given dataset. With the help of the defined function Distance(·), we could find that, compared with line segmentations *l*_*p*_0_*p*_1__, *l*_*p*_0_*p*_2__, and *l*_*p*_0_*p*_3__, the distance of *p*_0_ and *p*_4_, say *l*_*p*_0_*p*_4__, is the largest. So if *p*_5_ is the centroid point of triangle *p*_1_ *p*_2_ *p*_3_, then 3*l*_*p*_4_*p*_5__ is the centroid distance of *p*_0_. Therefore, the density around point *p*_0_ is 1/(3*l*_*p*_0_*p*_5__). Considering the correlation between centroid distance and density, we will use the value of centroid distance directly to describe density in the remainder of this paper.

**Figure 3:**
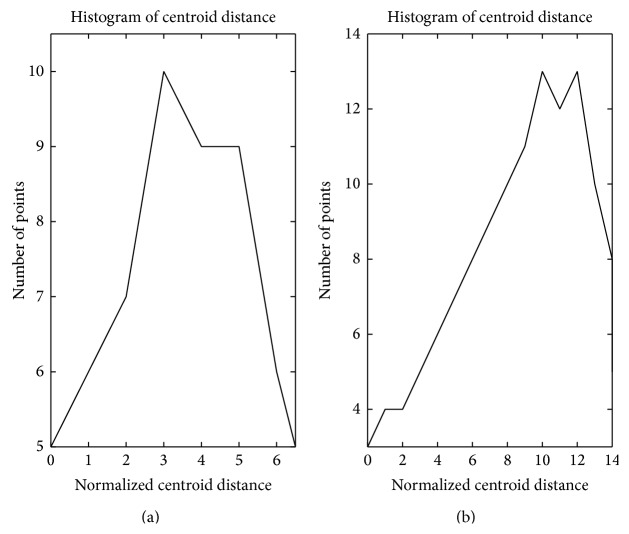
Centroid distance histogram of two homogeneous clusters.

**Figure 4:**
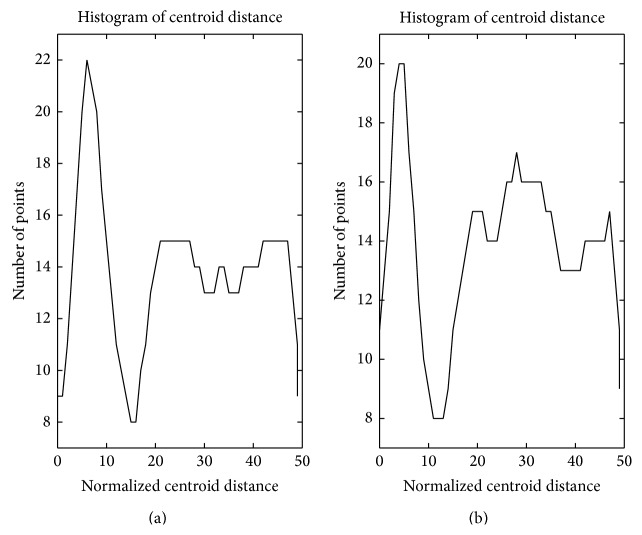
Centroid distance histogram of two heterogeneous clusters.

Based on the analysis above, the points' densities in cyan circle-shaped cluster and blue circle-shaped cluster in Figure 1 are analysed as Figures 3(a) and 3(b); the points' densities in red forks cluster and green forks cluster are analysed as Figures 4(a) and 4(b). The horizontal axis in these figures represents normalized centroid distance, while the vertical axis represents the number of points. Comparing Figure 4 with Figure 3, some law could be found. The density distribution of cyan circle-shaped cluster and blue circle-shaped cluster, which are homogeneous, has only one peak, as what is shown in Figure 3. In contrast, there are at least two apparent peaks on the density distribution curve of red forks and green crosses clusters, whose densities are nonhomogeneous, as what is shown in Figure 4. Therefore, an analogy can be drawn that the centroid distance distribution curve of a given cluster would have more than one peak if heterogeneity exists. Furthermore, based on this analogy, the centroid distance values corresponding to the valleys on centroid distance distribution curve which has more than one peak could be seen as a new isolation criterion.

### 2.3. Centroid Distance Isolation Criterion Based on Nonhomogeneous Density

In order to identify different density distributions within a cluster, we assume that its centroid distance distribution obeys Gaussian Mixture Models (GMMs) as long as heterogeneity exists. More specifically, if there are *n* valleys on density distribution curve, then, for point *x*_*i*_, *p*(Centroid_Distance(*x*_*i*_)) obeys a GMM consisting of *n* + 1 Gaussian distribution components, as shown in the following formula, in which

(2)∑i=1n+1πi=1,Nix ∣ μi,σi=12πσiexp⁡−12σix−μi2,(3)pCentroid_Distancexi=∑i=1n+1πiNiCentroid_Distancexi ∣ μi,σi.

**Figure 5:**
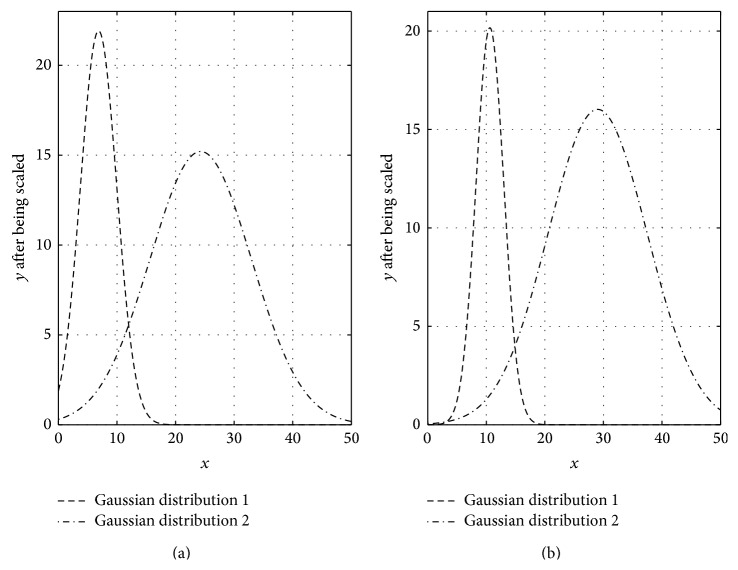
GMMs derived by EM algorithm from two heterogeneous clusters.

Based on the GMM assumption, we used EM algorithm to derive two sets of parameters *π*_*i*_, *μ*_*i*_, and *σ*_*i*_ for the red forks and green forks clusters in Figure 1. The results are shown in Figures 5(a) and 5(b), where the dashed-line curve represents high density area and the dashed-dot curve represents the other area. Therefore, the components of a GMM could be derived from a given cluster whose centroid distance distribution curve has at least one valley. Specifically, the *x* values of the intersection points of different Gaussian distributions in a GMM could be seen as isolation criterion.

In terms of efficiency, the complexity of EM algorithm depends on the number of iterations and the complexity of E and M step, which is seriously related with cluster size. In order to guarantee the efficiency of isolation criterion's computation, we designed a more simple algorithm which could reduce the computational complexity to *O*(*n*), where *n* is the number of points in a given cluster. For the next paragraph, we will describe the thought of simplification.

**Figure 6:**
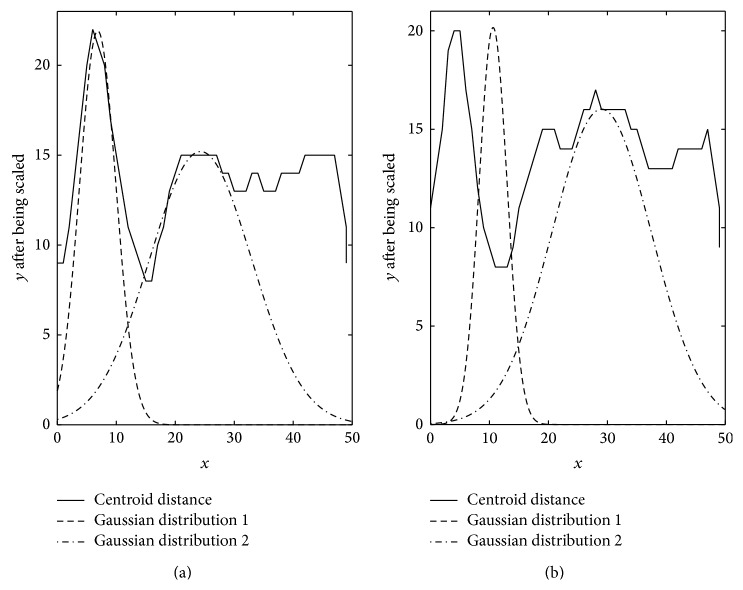
Comparison of GMM and centroid distance distribution curve.

**Figure 7:**
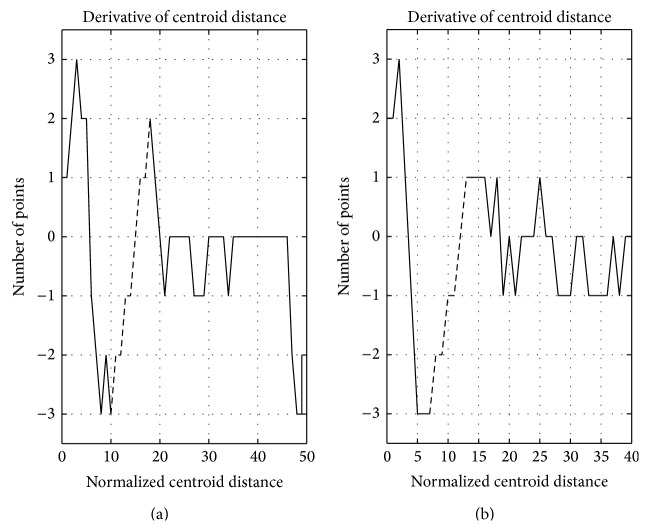
Centroid distance derivative of two heterogeneous clusters.

Through the observation of Figure 6, which demonstrates a comparison of GMM and centroid distance distribution curve, we could find that the *x* values of the lowest point of the valley on centroid distance distribution curve and the intersection point of two Gaussian distributions are almost identical. So the task of identifying a GMM can be converted into identifying the valleys on a centroid distance distribution curve. Intuitively, if a valley is deep enough, the corresponding centroid distance of the lowest point will be a good partitioning value. The concept of derivation is then utilized to reflect this intuition here.  Figure 7 illustrates the derivative of the centroid distance distribution curves in Figure 6. The derivative segmentation corresponding to a peak-valley-peak segmentation on a density distribution curve must satisfy two requirements. The first is that it has to cross zero point of vertical axis, which means that there is indeed a valley on centroid distance distribution curve there. On the premise of meeting this requirement, the derivation segmentation still needs to be long enough, which means that the valley has enough depth to be a good isolation value. The dashed-line segmentations in Figure 7 satisfy these two requirements, and the corresponding centroid distance values are 6 and 8, which are nearly identical with the *x* values of the intersection points of two Gaussian distributions in Figure 5.

Based on the analysis above, a nonhomogeneous density detection algorithm is proposed to carry potential partitions within a given cluster. This algorithm first uses crossing-zero index to filter optional partitioning values from all points and then measures the angles on either side of this point on centroid distance distribution curve to evaluate the significant level of the isolation criterion. A schematic description is as shown in Algorithm 1.

**Figure 8:**
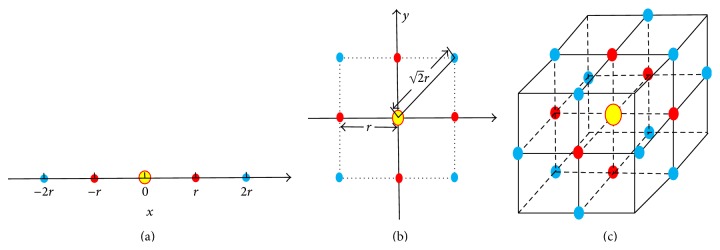
Uniformly distributed points in one-, two-, and three-dimensional space.

In our nonhomogeneous density detection algorithm, one parameter *n*, which is the number of points used to calculate centroid distance, still needs to be decided. In order to give a determination policy of *n*, let us consider three concrete examples in Figures 8(a), 8(b) and 8(c), which represent uniformly distributed points in one-, two-, and three-dimensional space, respectively. Uniformly distributed points means, for a given point, there exist two nearest equidistant points on every dimension. In our examples, Euclidean distance is used and the value of nearest equal distance is *r*. Further investigation tells us that the change of distance from a given point is not continuous but discrete. In Figure 8, for the central yellow point, the first-level nearest points are marked in red, and the second-level nearest points are marked in blue. The three subfigures are summarized in Table 1, based on which formula (4) is put forward to calculate the number of *k*-level nearest points in *d*-dimensional space (*k* ≤ *d*). More specifically, when *k* equals 1, formula (4) is reduced to be the number of first-level nearest points, which is 2*d*. We believe that the number of first-level nearest points is sufficient for centroid distance computation in uniformly distributed dataset. In reality, however, data can hardly be uniformly distributed, so in order to guarantee the availability of centroid distance to reflect nonhomogeneous density we multiply the first-level nearest points' number by 2. Formula (5) finally gives the policy to determine *n* in nonhomogeneous density detection algorithm according to the dimension of data set:

(4) n=Cdk2k,

(5) n=4d.

### 2.4. The Integration of Dissimilarity Increment and Centroid Distance Criteria

**Figure 9:**
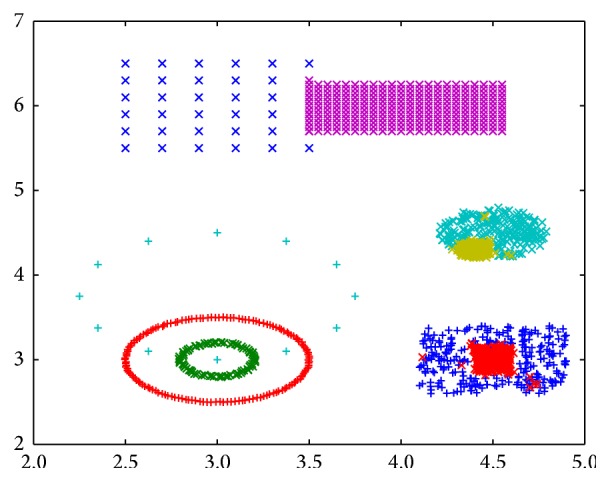
Result generated by our LASS algorithm.

Applying nonhomogeneous density detection algorithm after using dissimilarity increments clustering method, in other words, taking dissimilarity increments and centroid distance as an isolation criterion successively, a new clustering algorithm named localized ambient solidity separation algorithm (LASS) is developed, and the clustering result is obtained. Just as demonstrated in Figure 9, except for the perfect partition of naturally isolated clusters, their internal structure has also been explored and points are partitioned further if necessary. The yellow, red, and green clusters in Figure 1 are divided into two subclusters further according to their nonhomogeneous density distribution. Therefore, our LASS algorithm can handle clusters of arbitrary shape which are isolated, adjacent, overlapping, and under background noise. Moreover, compared with the traditional notation of density, which is the number of points in unit Euclidean volume, our proposed centroid distance isolation criterion works well in high-dimensional space; actually it is even more sensitive as dimension increases. Also, compared with direct similarity, centroid distance isolation criterion takes into account the surrounding context of each point by using its* n*'s nearest points and depends on the histogram distribution instead of the exact absolute value of similarity. So it can automatically scale according to the density of points. All in all, integrated dissimilarity increments and centroid distance isolation criteria together, our LASS algorithm can achieve broader applicability, especially on the dataset with high dimension and diverse distribution shape.

## 3. Computer User Segmentation

In this section, our proposed LASS algorithm is applied on computer users dataset which contains their demographic and behaviour information. To accomplish this, we first cleaned the raw data and extracted 7 features to characterize computer users. Then the cleaned data is normalized and a dissimilarity measurement is defined. On the basis of these, the original dissimilarity increments clustering algorithm and our LASS algorithm are applied on the dataset, respectively. The clustering processes are analysed and the effectiveness of results is verified. At last, the segmentation result of computer users is analysed and summarized.

### 3.1. Data Cleaning and Features Selection

The raw data provided by CNNIC contains two kinds of information. They are 1000 computer users' personal attributes and their computer using log files. Specifically, personal attributes include a volunteer's gender, birthday, education level, job type, income level, province of residence, city of residence, and type of residence, while computer using log files record these 1000 volunteers' computer interaction behaviours in 7 days, including start time, end time, websites browsing history, and programs opening history.

Although many features could be extracted from raw data, we focus our attention on volunteers' natural attributes as persons and their fundamental behaviours' statistical indicators but ignore environmental and geographic factors, such as job type, province of residence, city of residence, and residence type. The reason behind this is that we regard Internet as a strength which has broken down geographic barrier. Therefore, we assume that environmental and geographic factors are no longer crucial influence factors in Internet world. From this point of view, we extracted 7 features to profile computer users. Taking the *i*th computer user *u*_*i*_ as a concrete example, these extracted features are described in Table 2. The data of volunteers whose value of Times(·) is less than 4 are cleared out, and 775 sample data are left.

### 3.2. Data Normalization and Dissimilarity Measurement

Data normalization is needed before applying our LASS algorithm. The reason to do so is that similarity measurement is usually sensitive to differences in mean and variability. In this paper, two kinds of normalization are used, as expressed in formulas (6) and (7), respectively. In formula (6), *m*_*j*_ and *s*_*j*_ are the mean and standard deviation of feature *j*. Through this transformation, feature *j* will have zero mean and unit variance. While in formula (7), function Rank(·) returns the ranked number of *x*_*ij*_^*∗*^ in feature *j* data sequence. Therefore the transformed data will have a mean of (*n* + 1)/2 and a variance of (*n* + 1)[(2*n* + 1)/6 − (*n* + 1)/4], where *n* is the number of data. Related study has shown that, on the performance of clustering, formula (7) outperforms formula (6); particularly in hierarchical clustering methods, formula (7) is more robust to outliers and noise in dataset [28]:

(6) xij=xij∗−mjsj,

(7) xij=Rankxij∗.

In this paper, for continuous variable's normalization such as bootDuration(·) and visitingDuration(·), formulas (7) and (6) are used successively, while for discrete variable's normalization such as Gender(·), Age(·), and Edu(·) only formula (6) is used.

After normalization, a dissimilarity index is defined to measure the distance between different data. As formula (8) shows, it is a form of 1-norms' sum, where *f*_*in*_ stands for the value of *i*th data's *n*th feature:

(8) $$\begin{array}{c} Dissimilarity\;\left(\;u_{i},u_{j}\;\right)=\overset{7}{\underset{n=1}{\sum }}\left|\;f_{in}-f_{jn}\;\right|. \end{array}$$

### 3.3. Computer Users Segmentation Process

Our proposed LASS algorithm is applied for the segmentation of computer users in this section. The whole segmentation process consists of two parts. Part I is the dissimilarity increments based clustering strategy (for details please refer to Section 3 in [27]), which aims to find natural isolated clusters; part II is our proposed centroid distance based clustering strategy (for details please refer to Section 2.3 in this paper), whose goal is to explore the internal structure of every cluster generated by part I and identify potential subclusters that are adjacent, overlapping, and under background noise.

**Figure 10:**
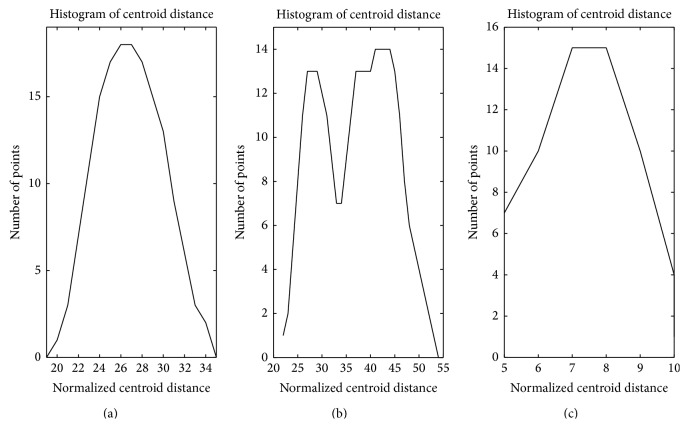
Centroid distance histogram of three clusters.

**Figure 11:**
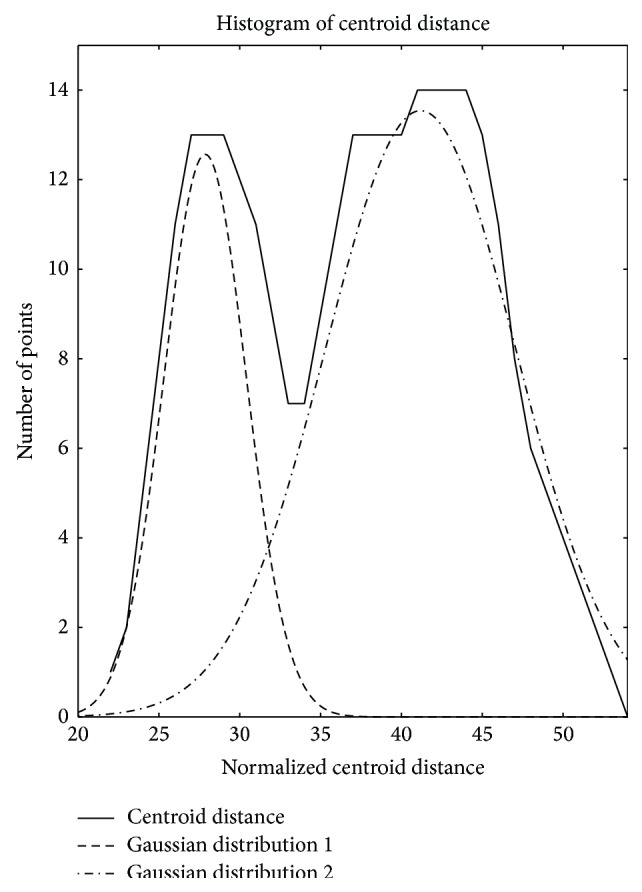
Comparison of GMM and centroid distance distribution curve.

The clustering process is partly shown in Figure 10, where three representative clusters obtained in part I strategy are chosen to be demonstrated. Further exploration is carried out by part II strategy of LASS algorithm, and a partition valley is found in cluster 2, as shown in Figure 10(b). Next, the horizontal axis value of the lowest point on this valley can be acquired as a further isolation criterion, based on which cluster 2 will be divided into two subclusters.  Figure 11 shows a comparison of the GMM generated by EM algorithm and centroid distance distribution curve of cluster 2. Despite the differences between these two graphs' shapes, the acquired two isolation criteria are nearly the same, which validates our simplification of GMM's computation.

### 3.4. Segmentation Results Analysis and Discussion

The segmentation results generated by the original dissimilarity increments method and our LASS algorithm are demonstrated in Tables 3 and 4. These two tables list the prototypes summarized from the obtained clusters. As it is shown, the sixth cluster in Table 3 is divided into two subclusters, the sixth and seventh cluster in Table 4. The reason of this further partition, as analyzed in Section 3.3, is the existence of a deep enough valley on cluster 6's centroid distribution curve (as shown in Figure 10(b)), which implies the existence of two different density areas within cluster 6 in Table 3.

To understand this process, some investigation should be made about the relationship between Tables 3 and 4. In Table 3, cluster 6 is the largest group of all clusters, whose gender proportion is almost 50%. However, an intuitive sense of behavior tells us that behavior mode should be seriously affected by people's gender. This intuition is proved by the first 5 clusters in Table 3 to some extent, in which gender proportion is 100% male. The reason why cluster 6 has not been divided further apart by the dissimilarity increments clustering method is that there may exist much touching areas in high-dimensional space of cluster 6, under which situation the dissimilarity increments clustering method does not work anymore. While our proposed centroid distance based nonhomogeneous density detection algorithm has found that there still exist two potential subgroups within cluster 6 in Table 3, which are identified as clusters 6 and 7 in Table 4, these two clusters are different in gender, age, and computer using behaviors. Cluster 6 is almost totally composed of women, who spend less time on computer and websites browsing, while in cluster 7 men are twice as much as women who are older than people in cluster 6 and spend much more time on computers, especially on browsing.

In order to quantify the overall effectiveness of our LASS algorithm, a between group sum of dissimilarities (SDB) is calculated as formula (9), which is the sum of the dissimilarity between a cluster centroid, *c*_*i*_, and the overall centroid, *c*, of all the data. In this formula, *K* is the number of clusters and *n*_*i*_ is the number of points in cluster *i*. The higher the total SDB is achieved, the more adjoint the identified clusters are. So it could be used to measure the effectiveness of a clustering method. The total SDB of the original dissimilarity increments clustering method and our LASS algorithm on the given dataset are shown in Table 5. Obviously, our LASS algorithm achieves larger total SDB, more specifically 30% larger; thus it fits for the given computer user dataset better.

In terms of the evaluation of individual clusters, silhouette coefficient is used here, whose value varies between −1 and 1. A positive value of silhouette coefficient is desirable. As Table 6 shows, the silhouette coefficient value of cluster 6 in Table 3 is negative, which implies that the inside cohesion and outside separation of the cluster are not good. So cluster 6 in Table 3 could not be seen as a typical cluster, while through our LASS algorithm cluster 6 in Table 3 is identified as two individual clusters, one of whose silhouette coefficients is positive. So as to cluster 7, whose silhouette coefficient is still negative, we guess that it belongs to some kind of background noise. This will be discussed later. As for cluster 6 in Table 4, we believe that it is a typical prototype of Chinese female computer users, which has not been revealed in Table 3. Therefore, compared with the original dissimilarity increments clustering method, our LASS algorithm can gain more knowledge and understanding from computer user dataset:

(9) $$\begin{array}{c} \text{Total SDB}=\overset{K}{\underset{i=1}{\sum }}n_{i}\;\text{Dissimilarity}\left(\;c_{i},c\;\right). \end{array}$$

Further, Kruskal-Wallis H Test is applied on the clusters in Table 4 to test the difference between two or more clusters of a given dimension. As a nonparametric test method, Kruskal-Wallis H Test is typically used to determine if there are statistical significance differences between two or more groups of an independent variable. The results are shown in Tables 7 and 8. In the hypothesis tests of Table 7, original hypothesis is that the distributions of a given variable in all 7 clusters are identical, and alternative hypothesis is that the distributions of a given variable in all 7 clusters are not identical. While in the hypothesis tests of Table 8, original hypothesis is that the distributions of a given variable in a given pair of clusters are identical, and alternative hypothesis is that the distributions of a given variable in a given pair of clusters are not identical. The *p* values are listed and marked by star if they are bigger than 0.05, which means accepting the original hypothesis and rejecting the alternative one. For the cases in which *p* value is below 0.05, the smaller the *p* value is, the more statistically significant the variable's difference is. In Table 7, all of the *p* values are below 0.002, which means, for any given variable, its distributions are extremely different among the seven clusters in Table 4. Therefore we can draw the conclusion that these seven variables perform well in identifying different groups of computer users. While in Table 8 *p* value changes a lot according to the given pair of clusters and variable. The significance of these seven variables to distinguish different pair of clusters will be discussed one by one combined with Table 9, which reveals the detailed demographic and computer interaction behaviours characteristics of the obtained seven computer users clusters.

Segmentation results will be analysed from the perspective of variables with the help of Table 8 and Tables 9 and 4, and significant characteristics will be pointed out. For the variable of gender, Table 8 tells us that its distributions in the first five segments are identical, which is proved to be 100% male in Table 9. The most significant difference of gender lies among segments 1–5, segment 6, and segment 7, which represents male groups, female group, and mix-gender group, respectively. For the variable of age, Table 8 reveals that its distribution among segments 4–7 could be seen as identical; the main difference happens between the first three segments. Combined with Tables 9 and 4, we could find that segment 2 consists of the youngest members, whose age is around 24. Segment 1 is a little bit elder group, whose average age is around 28. While segment 3 is a middle-aged group with an average age of 41, they are much older than other segments. So as to the variable of education level, it discriminates different segments well. Its distribution in segments 2 and 5 could be seen as identical that has the highest education level, bachelor degree, while the people from segment 4 have the lowest education level. Other segments differ from one another. For the variable of income level, segment 1 earns the highest income, while segment 2 earns the lowest one. The income level of segments 3 and 5 could be seen as identical, so it is with segments 4 and 6. And the former two's income is lower than the latter two's. In the terms of computer using frequency, the segments could be divided into two groups; they are segments 1, 2, and 7 and segments 3–6. The former group uses computer more frequently. As for the variable of computer using time, it discriminates segments 1 and 4 well that spend the most and the least time on computer, respectively, while for the remaining 5 segments no significant difference exists among their computer using time. For the last variable, website browsing time, its distribution in segments 2, 3, 4, and 6 could be seen as identical; difference mainly lies among segments 1, 5, and 7. Specifically, segment 1 spends the least time on website browsing, while segment 5 spends the most, and the browsing time of segment 7 falls in between segment 1 and segment 5.

Based on the analysis above, the 7 segments obtained by our LASS algorithm are summarized and discussed below, respectively.

**Category 1 (little-browsing group).**

This group is entirely composed of young men, who received a high education level and earn a decent income. The most significant feature of the people in this group is that although they spend the most time on computers compared with other groups, they seldom visit webpages. We guess that, for this group of people, the computer interaction behaviours mainly happen in workplace or public, where personal browsing is not encouraged.

**Category 2 (little-income group).**

This group is composed of the youngest people, who are purely male and have the highest education level. The most significant feature of this group of people is that they have the same income level, which is no income. Additionally, they spend relatively more time on computers and browsing websites. We guess that the main body of this group is college students in progress, who have lots of free time but no source of revenue.

**Category 3 (high-income group).**

This group of people is entirely middle-aged men. The most significant feature of the people in this group is the highest income they earn. Besides, they spend relatively less time on computer interaction in terms of both using frequency and total browsing time. We guess that, for the middle-aged men in this group, most of whom have not received a higher education, computers or Internet is not so necessary in their daily life.

**Category 4 (low-education group).**

This group is entirely composed of young men, whose age is older than Categories 1 and 2. The most significant feature of the people in this group is their low-education level, the average of which is senior school, ranging from junior school to junior college. Moreover, they earn a medium level income and get smaller values on every computer interaction index. We guess that this group of people is mainly engaged in jobs independent of computers.

**Category 5 (much-browsing group).**

The structure of this group is very similar to Category 4, except for the higher education they received, say, bachelor degree. As it is shown, people in this group earn more; we guess that education difference may account for this. Also, compared with other categories, especially Category 4, this group of people spends much more time on browsing websites. We guess that the main job types of this group could be intellectual work; thus they have close access to online computers.

**Category 6 (young-women group).**

Female accounts for nearly 100% in this group, which is the only case in these 7 categories. However, from computer interaction aspects, say, using frequency and browsing time, this group is very similar to Category 4. So we guess that these two groups of people have similar type of job or similar working circumstance. Moreover, although these young women have a higher education level than men in Category 4, they do not earn a better salary. We guess that this phenomenon may be due to the lack of career experience and gender discrimination.

**Category 7 (noise group).**

This category is the only gender mixed group, in which men are twice as much as women. However, in terms of age, education level, and income level, this category shows no significant difference compared with total population. And as for the variables of computer using frequency, computer using time, and website browsing time, their variances are fairly large, even bigger than the overall variances. So due to the dispersed distribution of this category on every dimension, we believe that it is a noise group.

## 4. Conclusion

In this paper, we proposed a new clustering algorithm named localized ambient solidity separation (LASS) algorithm. This algorithm is built on a new isolation criterion called centroid distance, which is used to detect the nonhomogeneous density distribution of a given cluster. The proposed isolation criterion is based on the recognition that if there exist nonhomogeneous densities within a cluster, then partitions should be carried out. The intuition behind this recognition is GMM assumption of the points' centroid distance value in a cluster. EM algorithm was used to derive the components and parameters of a GMM. Additionally, in order to make the algorithm more efficient, we designed a nonhomogeneous density detection algorithm to reduce computation complexity to *O*(*n*), where *n* is the number of points for clustering. Moreover, the parameter determination policy of nonhomogeneous density detection algorithm is investigated. Finally, we integrated our designed nonhomogeneous density detection algorithm, as a follow-up mechanism, with the original dissimilarity increments clustering method, and developed LASS algorithm. It is demonstrated that, compared with the original dissimilarity increments clustering method, our LASS algorithm not only can identify naturally isolated clusters but also can identify the clusters which are adjacent, overlapping, and under background noise.

Additionally, in order to evaluate the performance of LASS algorithm in practice, we applied it on the computer user dataset, which contains 1000 computer users' demographic and behaviours information, comparing with the result got from the original dissimilarity increments clustering method. The segmentation results show that one of the clusters generated by the dissimilarity increments clustering method is further divided into two subclusters by our LASS algorithm. The comparison of total SDB and silhouette coefficient validates the rationality of this further partition. The discussion and analysis of segmentation results are made and prove that our LASS algorithm can gain more knowledge and understanding from dataset with high dimensionality and diverse distribution shapes, like computer user dataset.

There are some future directions to explore from this paper. First, the GMM assumption of centroid distance value can be further investigated and tested among more distributions, such as Gaussian and exponential. Second, our proposed centroid distance isolation criterion could be integrated with other traditional clustering methods, either partitional or hierarchical; more strengths and weaknesses could be pointed out and analysed. Third, the centroid distance based clustering strategy in our LASS algorithm relies on the histogram distribution of centroid distance values; therefore if the number of points in one cluster is too small, this clustering strategy may not work effectively any more. This drawback should be given enough attention and further investigated.

## Figures and Tables

**Algorithm 1:**
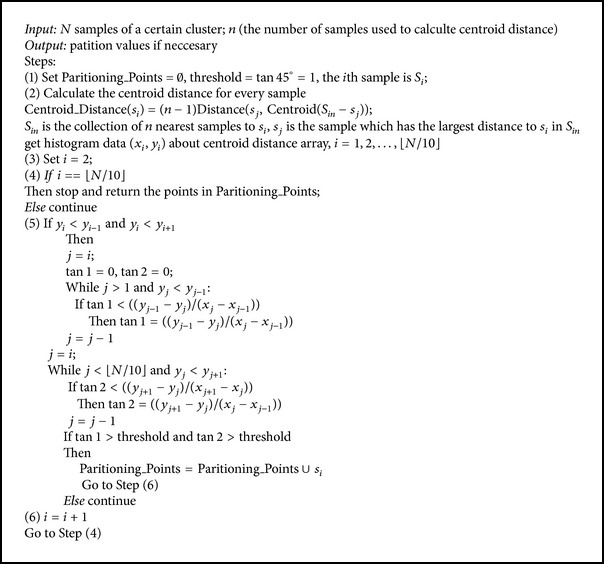


**Table 1:** First- and second-level nearest points in uniformly distributed space.

Dimensions	One	Two	Three
First-level nearest points	2	4	6

Second-level nearest points	2	4	12

**Table 2:** Description of computer users features.

Variables	Descriptions
Gender (*u*_*i*_)	The gender of *u*_*i*_, discrete variable 1 stands for male; 0 stands for female

Age (*u*_*i*_)	The age of *u*_*i*_, discrete variable between 10 and 70

Edu (*u*_*i*_)	The education level of *u*_*i*_, discrete variable 0: below primary school 1: junior school 2: senior school 3: junior college 4: bachelor degree 5: others

Income (*u*_*i*_)	The monthly income level of *u*_*i*_, discrete variable 0: no income 1: below 500 Yuan 2: 501–1000 Yuan 3: 1001–1500 Yuan 4: 1501–2000 Yuan 5: 2001–3000 Yuan 6: 3001–5000 Yuan 7: 5001–8000 Yuan 8: 8001–12000 Yuan 9: others

Times (*u*_*i*_)	Boot times of *u*_*i*_'s computer, discrete variable

Booting Duration (*u*_*i*_)	The duration of *u*_*i*_ using computer, continuous variable

Brows Duration (*u*_*i*_)	The duration of *u*_*i*_ browsing websites, continuous variable

**Table 3:** Results generated by dissimilarity increments clustering method.

Segment	Size	Gender	Age	Education level	Income level	Computer using frequency (times/week)	Computer using time (hours/week)	Website browsing time (hours/week)
1	24	Male: 100% Female: 0%	28	Junior college	2001–5000 Yuan	6.7	67.5	0.6

2	35	Male: 100% Female: 0%	24	Bachelor degree	0–500 Yuan	6.5	44.8	4.4

3	58	Male: 100% Female: 0%	41	Junior college	3001–5000 Yuan	5.7	44.7	2.9

4	70	Male: 100% Female: 0%	32	Senior school	2001–3000 Yuan	6	33	3.1

5	185	Male: 100% Female: 0%	32	Bachelor degree	2001–5000 Yuan	5.9	39	6.7

6	352	Male: 42% Female: 58%	32	Junior college	1501–3000 Yuan	6.5	42.1	5.1

**Table 4:** Results generated by our LASS algorithm.

Segment	Size	Gender	Age	Education level	Income level	Computer using frequency (times/week)	Computer using time (hours/week)	Website browsing time (hours/week)
1	24	Male: 100% Female: 0%	28	Junior college	2001–5000 Yuan	6.7	67.5	0.6

2	35	Male: 100% Female: 0%	24	Bachelor degree	0–500 Yuan	6.5	44.8	4.4

3	58	Male: 100% Female: 0%	41	Junior college	3001–5000 Yuan	5.7	44.7	2.9

4	70	Male: 100% Female: 0%	32	Senior school to junior college	2001–3000 Yuan	6	33	3.1

5	185	Male: 100% Female: 0%	32	Bachelor degree	2001–5000 Yuan	5.9	39	6.7

6	136	Male: 0.7% Female: 99.3%	30	Junior college to bachelor degree	1501–3000 Yuan	5.9	37.8	3.1

7	216	Male: 68.1% Female: 32.9%	33	Junior college	1001–2000 Yuan	6.9	44.8	6.3

**Table 5:** Total SDB of two clustering methods.

Method	Dissimilarity increments clustering method	Our LASS algorithm
Total SDB	853	1109

**Table 6:** The silhouette coefficients of clusters.

Clusters	Cluster 6 in Table 3	Cluster 6 in Table 4	Cluster 7 in Table 5
Silhouette coefficient	−0.34	0.02	−0.41

**Table 7:** *p* values of features among all clusters.

Variables	Gender	Age	Education level	Income level	Computer using frequency	Computer using time	Website browsing time
*p* value	<0.002	<0.002	<0.002	<0.002	<0.002	<0.002	<0.002

**Table 8:** *p* values of features between two pairs of clusters.

Variables	Pair of segments						
	1-2	1-3	1-4	1-5	1-6	1-7	2-3
Gender	>0.5^*∗*^	>0.5^*∗*^	>0.5^*∗*^	>0.5^*∗*^	<0.002	<0.002	>0.5^*∗*^
Age	<0.005	<0.002	>0.05^*∗*^	<0.05	>0.2^*∗*^	>0.05^*∗*^	<0.002
Education level	<0.002	>0.5^*∗*^	<0.002	<0.002	>0.05^*∗*^	>0.5^*∗*^	<0.002
Income level	<0.002	>0.2^*∗*^	>0.1^*∗*^	>0.5^*∗*^	>0.05^*∗*^	<0.002	<0.002
Computer using frequency	>0.2^*∗*^	<0.002	<0.005	<0.002	<0.002	>0.1^*∗*^	<0.005
Computer using time	<0.002	<0.002	<0.002	<0.002	<0.002	<0.002	>0.5^*∗*^
Website browsing time	<0.002	<0.05	<0.01	<0.002	<0.05	<0.002	>0.1^*∗*^

Variables	Pair of segments						
	2-4	2-5	2-6	2-7	3-4	3-5	3-6

Gender	>0.5^*∗*^	>0.5^*∗*^	<0.002	<0.002	>0.5^*∗*^	>0.5^*∗*^	<0.002
Age	<0.002	<0.002	<0.002	<0.002	<0.002	<0.002	<0.002
Education level	<0.002	>0.5^*∗*^	<0.002	<0.002	<0.002	<0.002	<0.005
Income level	<0.002	<0.002	<0.002	<0.002	<0.005	>0.1^*∗*^	<0.002
Computer using frequency	<0.05	<0.005	<0.002	>0.5^*∗*^	>0.2^*∗*^	>0.2^*∗*^	>0.5^*∗*^
Computer using time	<0.01	>0.2^*∗*^	>0.1^*∗*^	>0.5^*∗*^	<0.002	>0.1^*∗*^	<0.05
Website browsing time	>0.2^*∗*^	>0.05^*∗*^	>0.05^*∗*^	>0.5^*∗*^	>0.5^*∗*^	<0.002	>0.5^*∗*^

Variables	Pair of segments						
	3-7	4-5	4-6	4-7	5-6	5-7	6-7

Gender	<0.002	>0.5^*∗*^	<0.002	<0.002	<0.002	<0.002	<0.002
Age	<0.002	>0.5^*∗*^	>0.1^*∗*^	>0.5^*∗*^	<0.02	>0.2^*∗*^	>0.1^*∗*^
Education level	>0.2^*∗*^	<0.002	<0.002	<0.002	<0.002	<0.002	<0.002
Income level	<0.002	<0.05	>0.5^*∗*^	<0.02	<0.002	<0.002	<0.02
Computer using frequency	<0.002	>0.5^*∗*^	>0.2^*∗*^	<0.005	>0.5^*∗*^	<0.002	<0.002
Computer using time	>0.5^*∗*^	<0.01	>0.05^*∗*^	<0.002	>0.2^*∗*^	>0.1^*∗*^	<0.05
Website browsing time	<0.005	<0.002	>0.2^*∗*^	<0.02	<0.002	<0.05	<0.002

**Table 9:** Demographic and behaviour description of computer user segmentations.

Demographic and computer interaction behaviours characteristics	Segment 1	Segment 2	Segment 3	Segment 4	Segment 5	Segment 6	Segment 7	Total
Gender								
Male	100	100	100	100	100	0.7	68.1	71.8
Female	0	0	0	0	0	99.3	31.9	28.2
Age								
10~20	0	0	0	0	0	2.2	12.0	4.0
20~25	4.2	68.6	6.8	14.3	6.4	12.5	17.6	14.6
25~30	62.5	22.9	3.4	24.2	34.1	41.9	16.2	27.2
30~35	33.3	8.6	13.8	28.6	27.6	21.3	16.2	21.2
35~40	0	0	15.5	22.9	16.2	14.7	10.2	13.4
40~50	0	0	37.9	10	13.5	5.9	18.1	14.0
50~60	0	0	20.6	0	2.2	1.4	8.3	5.0
60~70	0	0	1.7	0	0	0	1.4	0.6
Education level								
Below primary school	0	0	0	0	0	0	1.4	0.4
Junior school	0	0	1.7	2.9	0	2.2	14.4	5.1
Senior school	0	0	25.9	74.3	0.5	19.9	26.9	21.1
Junior college	100	20	55.1	22.9	26.5	33.1	19.9	29.8
Bachelor degree	0	71.4	17.2	0	73.0	41.2	28.7	39.8
Others	0	8.6	0	0	0	3.7	8.8	3.7
Income level								
No income	0	91.4	0	0	0	5.1	24.1	12.6
Below 500 Yuan	0	5.7	0	0	0	0.7	3.2	1.4
501–1000 Yuan	0	2.9	1.7	0	1.6	2.9	4.6	2.6
1001–1500 Yuan	0	0	5.2	11.4	4.3	5.9	9.7	6.6
1501–2000 Yuan	12.5	0	10.3	22.9	10.8	17.6	8.3	12.0
2001–3000 Yuan	29.2	0	24.1	24.3	28.6	32.4	16.2	12.5
3001–5000 Yuan	45.8	0	20.7	32.9	39.5	23.5	15.7	25.6
5001–8000 Yuan	12.5	0	17.2	8.5	11.9	6.6	8.3	9.4
8001–12000 Yuan	0	0	12.1	0	3.2	4.4	2.8	3.4
Others	0	0	0	0	0	0.7	6.9	2.9
Computer using frequency								
Mean	6.7	6.5	5.7	6.0	5.9	5.87	6.9	6.3
Variance	0.04	0.20	0.33	0.18	0.25	0.40	1.4	0.68
Computer using time								
Mean	67.5	44.7	44.7	33.0	39.4	37.8	44.8	41.7
Variance	0.23	0.88	1.31	0.31	0.70	0.83	1.31	0.99
Website browsing time								
Mean	0.64	4.43	2.88	3.12	6.7	3.1	6.3	4.95
Variance	0.43	0.89	0.89	0.80	0.83	0.96	1.01	0.99
